# Antireflux Endoluminal Therapies: Past and Present

**DOI:** 10.1155/2013/481417

**Published:** 2013-07-09

**Authors:** Kuo Chao Yew, Seng-Kee Chuah

**Affiliations:** ^1^Department of Gastroenterology and Hepatology, Tan Tock Seng Hospital, 11 Jalan Tan Tock Seng, Singapore 308433; ^2^Division of Hepatogastroenterology, Department of Internal Medicine, Kaohsiung Chang Gung Memorial Hospital, Chang Gung University College of Medicine, 123 Ta-Pei Road, Niao-Sung Hsiang, Kaohsiung 833, Taiwan

## Abstract

The basic principle of antireflux procedures employing endoscopic intervention aims to create a mechanical barrier to prevent primary pathophysiology in gastroesophageal reflux disease (GERD). We review, highlight, and discuss the past and present status of endoluminal therapy. Currently, there are 3 commonly employed anti-reflux endoluminal procedures: fundoplication or suturing techniques (EndoCinch, NDO, and EsophyX), intramural injection or implant techniques (enhancing lower esophageal sphincter (LES) volume and/or strengthening compliance of the LES-Enteryx and Gatekeeper), and radiofrequency ablation of LES and cardia. EndoCinch plication requires further study and modification of technique before it can be recommended because of durability issues. Esophynx, the transoral incisionless fundoplication, may reduce hiatal hernias and increase LES length. Preliminary studies have shown promising reduction in symptoms and medication use but evidence concerning safety and long-term durability is still pending. The safety issue with injection technique is the main concern as evident from the incidences of implant withdrawals after reported major adverse events. Future research with cautious monitoring is required before any new implant material can be recommended for commercial application. Radiofrequency ablation therapy is regaining popularity in treating refractory symptoms despite PPI use due to improved efficacy, durability, and safety after years of refinement of protocol.

## 1. Introduction

Gastroesophageal reflux disease (GERD) is a disease spectrum caused by regurgitation of stomach contents causing troublesome esophageal or extraesophageal symptoms as defined by Montreal definitions [1]. Either mild heartburn and/or regurgitation for at least 2 days per week or moderate to severe symptoms for at least one day per week qualifies as significant symptom-based diagnosis [2]. Phenotypical classifications of GERD are nonerosive reflux disease (NERD), erosive esophagitis (EE), and Barrett's esophagus (BE). Population-based study reported 15%–20% of the Western population experience reflux on a weekly basis which can lead to impoverishment of a country's economy and quality of life [3]. Dent et al. reported the prevalence of GERD in Sweden (15.5%), Italy (11.8%), China, Japan, Korea (3.4%–8.5%), and Taiwan (9%–24.6%), respectively [4]. Subanalysis shows that EE and hiatus hernia are more common in Europe than in Asia with the exception of Taiwan which reported similar EE prevalence as Europe. Over years, the prevalence of GERD is increasing by approximately 5% annually amongst the American population and other countries in the West such as Western Europe and Scandinavia [5]. Furthermore, the prevalence of GERD is also increasing in Asian countries [6]. Taiwan, with a population consisting mainly of Chinese ancestry, has one of the most published data on GERD. Yeh et al. reported 14.5% of prevalence in 1991 [7] only to be superseded by a prevalence of approximately 25%-26% from 2007 to 2011 [8, 9]. The social impact of GERD includes lost work days and increased public health cost. Medically, there is a risk of developing esophageal adenocarcinoma (EAC). Barrett's esophagus will develop in an incidence rate of 0.5% per year and Barrett's esophagus is a risk for esophageal adenocarcinoma (EAC) or of a 400-fold increased risk of EAC [10]. GERD increases the risk of EAC by 8.6-fold [11].

GERD and its associated clinical manifestations present a diagnostic challenge. A third of patients with GERD present with atypical symptoms or in fact may be asymptomatic. Wong et al. reported extraesophageal presentation or atypical complaints such as asthma (4.8%), chronic cough (13%), and laryngeal disorder (10%) [12]. Some authors have suggested a trial of proton pump inhibitors (PPI) with a temporal improvement in symptoms as an indirect diagnostic method but the risk is that BE, RE, and even EAC may be missed [13]. 

PPI have been the most effective treatment for GERD but discontinuation of medical therapy is likely to lead to clinical relapse. Long-term PPI users and patients who are noncompliant with daily PPI dosage may run into problems such as refractory GERD, NERD, EE, and BE. Side effects of PPI use that are gaining more attention amongst physicians include an increased incidence of hip fracture in postmenopausal women, pneumonia, enteric infections, and drug-drug interactions with clopidogrel. With long-term PPI use, there is also the issue of compliance and financial health costs. Therefore, some patients may be more suited to other treatment options such as surgery or endoscopic intervention. Antireflux procedures either via surgery or endoscopic intervention aim to create a mechanical barrier to prevent primary pathophysiology in GERD. In this paper, we review, highlight, and discuss the past and present status of the endoluminal therapies.

## 2. GERD Pathophysiology

The pathophysiological mechanisms of GERD are transient LES relaxation (TLESR), low LES pressure, and GEJ anatomic distortions such as hiatal hernia [14]. Dysfunctional esophageal motility, impaired barrier function of LES, and gastric emptying in relation to meal intake may all lead to gastric content reflux. LES relaxation can be triggered by 3 main motor events: deglutitive inhibition during the swallowing process, secondary peristalsis from esophageal distention, and cardial distention mediated relaxation of LES. The third mechanism is transient TLESR with the sensory trigger point located distal to LES [15]. It is the total relaxation duration and not the frequency that contributes to this pathophysiology. Classical description involves relaxation of LES, esophageal shortening, and inhibition of crural diaphragm. A disruption in the musculature plane such as hiatal hernia will blunt the angle of His and impair the flap valve mechanism. The GERD condition is mostly accompanied by the formation of a gastric air pocket which can stimulate the acid sensing ion channel primarily located around the cardia leading to TLESR [15]. Often it is bile acid that causes more mucosal damage than gastric secretion. 

## 3. Rationale of Antireflux Endoluminal Therapy

PPI therapy to decrease acid output cannot provide a physical barrier or restore the LES function [16]. Relapses in esophageal and extraesophageal disease are also indications for more definitive treatment. Chen et al. reported that bile acid disrupts squamous epithelial barrier function by modulating TJ proteins, demonstrating the importance of the integrity of an antireflux barrier in addition to established acid suppressive therapy [17]. Some patients may not tolerate long-term medical therapy or be a candidate for surgery. Under such circumstances, endoluminal antireflux interventions may be a viable option. Antireflux endoscopic intervention aims to create a mechanical barrier to prevent the regurgitation of gastric contents into the esophagus. An appropriate high pressure zone at LES needs to be produced to aid the closure of diaphragmatic crural fibers and the His angle of the gastroesophageal junction. An anatomic-physiological flap valve at gastroesophageal junction can be created by antireflux barrier reconstruction of collar sling musculature at the cardia [18]. The antireflux barrier reconstruction procedure aims to reconstruct the acute angle of His by enveloping the distal esophagus to the proximal stomach mimicking an intragastric valve that will prevent regurgitation of food in the presence of intragastric and intra-abdominal pressure [19].

Hill and Kozarek demonstrated that the LES pressure gradient can be increased by suturing a flap valve (valvuloplasty) which can then be further enhanced with the posterior attachment of gastroesophageal junction [20]. With hiatus hernia, suturing the GEJ to a fixation point intra-abdominally at the preaortic fascia will have a similar effect. The presence of an intragastric mucosal ridge is far more important in determining antireflux effect rather than an increased LES pressure gradient. The valvular appearance is a good predictor of the reflux status. As there are limitations in endoscopic manipulation of the esophagus, careful patient selection with little or no hiatal hernia is important to determine the success of this approach [18]. In general, a large hiatal hernia (especially paraoesophageal) should be referred for laparoscopic or open surgery. Other contraindications for antireflux endoluminal therapy include patients with refractory symptoms despite maximum therapy, esophageal strictures, dysmotility and Barrett's esophagus, severe liver disease, portal hypertension, varices, and coagulopathy [18].

Indications for antireflux endoluminal therapy are refractory GERD, PPI intolerance, a desire to stop drug therapy with concerns of long-term side effects, concerns about laparoscopic antireflux surgery side effects such as dysphagia, gas bloat, and finally symptomatic GERD after fundoplication.

## 4. Antireflux Endoluminal Therapy

Development of the antireflux endoluminal therapy was an attempt at correcting GERD's pathophysiology by increasing the LES pressure, reducing the frequency of TLESR, antireflux barrier construction, attenuation of esophageal sensation against refluxate, and anatomical reconstruction improving the angle of His or cardia for flap valve creation [21]. The available antireflux endoluminal therapies can be divided into fundoplication or suturing techniques (EndoCinch, NDO, and EsophyX), intramural injection or implants techniques (Enteryx, Gatekeeper), and radiofrequency ablation of LES and cardia (Stretta system) (Table 1). Procedures such as Endocinch and Stretta RFA are safe outpatient procedures [21, 22].

### 4.1. Endoscopic Fundoplication or Suturing Techniques

Endoscopic fundoplication can be accomplished using the Endo-Cinch (C.R. Bard Inc., Murray Hill, NJ, USA), EsophyX (EndoGastric Solutions, Redwood City, CA, USA), and SRS endoscopic stapling (Medigus Ltd., Tel Aviv, Israel) systems [21]. The procedure may be used to improve LES tone, remodel GEJ, and alter lower esophageal length. This would reduce esophageal sensitivity and improve gastroesophageal flap valve grading. 

#### 4.1.1. EndoCinch

This landmark procedure was described by Swain and Mills in 1986 and subsequently approved in April 2000 by FDA. Till today it remains a popular option with well-studied research and is often compared to surgical fundoplication [23]. However, intermediate to long-term performance of Endocinch is considered poor with endoscopic ultrasound demonstrating loosening of sutures from lack of full thickness fundoplication and poor mucosa apposition with sutures even after enhanced modification [24, 25]. There is no marked improvement in its durability in the sham-controlled study [26]. There was no obvious reversal of esophagitis and no improvement in pH evaluation [26]. Complications such as mucosal tear and microperforation have been reported but the number was small. Moreover, the efficacy of EndoCinch was shown to be inferior to surgical fundoplication [27].

#### 4.1.2. NDO PLICATOR

In 2003, NDO Surgical Company, USA, designed a full-thickness suturing transmural plicator to address the weakness of EndoCinch. The pilot study conducted by Chuttani et al. proved the safety of the procedure in humans [28]. The device created a valve resembling partial fundoplication. Rothstein et al. reported an extended improvement in QoL and reduction in PPI usage at 5 years when compared to the sham study [29]. The time reduction for pH < 4 after full-thickness plication (FTP) suggested the successful creation of a more effective mechanical barrier. Jeansonne IV et al. reported a superiority of NDO FTP against radio frequency ablation (RFA) in an obese cohort and also in patients with major complaints of regurgitation [30]. Larger hiatus hernia (especially >2 cm) and loose cardia diameter resulted in failure of procedure [13]. Technique modification such as application of 2 plications at slight diagonal vector was superior to conventional methods [30]. However, this procedure had similar side effects to fundoplication such as dysphagia, dysphonia, and cough [23]. The device was retrieved from market in June 2008 due to the company's poor financial performance. However, there are other competitive devices such as Antireflux Device (ARD; Syntheon, Miami, FL, USA), the His-Wiz (Olympus, Center Valley, PA, USA), and the EsophyX (EndoGastric Solutions, Redmond, WA, USA) [25]. 

#### 4.1.3. EsophyX

EsophynX, the transoral incisionless fundoplication (TIF), was evaluated in 2006. It utilises suction and transmural fasteners for the application of an uninterrupted suture line at the base of LES and opposing gastroesophageal junction to the fundus, thus creating a neoesophageal valve of 2–6 cm (average 4 cm) and 230° in circumference (range 160–300°) and restoring the angle of His, closely resembling those of Nissen fundoplication products. There is no repeated device intubation requirement [23]. More importantly it can be used to reduce hiatal hernia up to 2 cm, which is often not possible with other antireflux endoluminal therapies [7]. Cadière et al. reported total hiatal hernia reduction and beneficial increment of LES length after procedure [31]. However, serious complications such as esophageal perforation and postoperative bleeding were reported [21]. 

### 4.2. Injection/Implantation Techniques

There were numerous attempts to create an antireflux barrier by a bulking effect at LES which included injection of bovine dermal collagen, Teflon, polymethylmethacrylate microspheres (Plexiglas), and polytetrafluoroethylene (Polytef) with no remarkable benefits [32]. Devises such as Gatekeeper Reflux Repair System (Medtronic Inc., Minneapolis, MN, USA) and Enteryx (Boston Scientific Corporate, Natick, MA, USA) were removed from the market because of unsatisfactory benefit from symptoms control or objective measurement of antireflux properties and various degrees of complications. Cicala et al. reported pharyngeal perforation in a patient resulting in mediastinitis or surrounding organ inflammation after error in injection techniques at early postmarketing phase in 2005 [33].

To date, the safety issue of injection techniques is still in the main concern [34]. Therefore, further research and observation are required before it can be recommended for commercial application. The major obstacle to injection techniques is that the implant material must meet the criteria of producing minimal inflammation to surrounding organs. 

### 4.3. Radiofrequency Ablation

Radiofrequency Ablation (RFA), Stretta system (Curon Medical, Fremont, CA, USA), was first introduced in 2000 [35]. It utilises an inflatable balloon-mounted device that introduces 4 electrodes at the LES with RFA energy delivered under controlled temperature to produce a coagulation inflammation, necrosis, and fibrosis. The RFA energy is emitted circumferentially, extending from 2 cm above to 1.5 cm below the gastroesophageal junction and an additional six sets below the cardia [35–37]. The RFA energy can induce neurolysis of LES vagal nerve which results in reduced frequency of TLESR, improvement of gastric emptying, and also increasing the gastric yield pressure level needed to cause reflux episodes [38, 39]. It can also reduce esophageal sensitivity, inducing remodeling of the compliance of gastroesophageal junction and hence increasing the LES resistance [40]. Abdel Aziz et al. reported that the total esophageal acid exposure was reduced after-procedure and sustained at 12th month of evaluation [37].

The Stretta system improves GERD symptoms and reflux control, alleviates heartburn, and reduces PPI requirement in nearly two-thirds of patients [41]. Reymunde and Santiago reported a 4-year followup with sustained improvements in symptoms, quality of life, and drug use after the procedure [42]. The efficacy was again demonstrated by Dughera et al. which showed that 72.3% of patients remained PPI free at month 48 [39]. Most of the antireflux endoluminal procedures do not alter the esophageal acid exposure and fail to demonstrate reversibility of the esophagitis which is the early manifestation of BA and EAC development. The Stretta is able to improve the severity of esophagitis [43]. 

 Over the years, the Stretta technology has refined the recommended dosage to avoid serious complications. The early Stretta technique made 112 lesions at gastroesophageal junction resulting in some cases of esophageal perforation. A technical modification by Aziz group with the total number of lesions made reduced to 56 per session with a double-dose RFA energy delivered 4 months apart has been proven to be less harmful than a single vigorous dose with double the efficacy [37]. The adverse events of the Stretta procedure are mostly mild and transient such as transient chest discomfort (26.7%), fever (7.1%), and dysphagia (7.1%) [41]. The paradoxical adverse event of delayed gastric emptying is variable which could be due to the effect of double-dose RFA energy causing vagal nerve damage at gastric fundus which results in gastroparesis. Other significant complications reported by United States Food and Drug Administration are bleeding, mucosal injuries, aspiration, and effusion [44].

 As this procedure can be done easily in an outpatient setting, the Stretta system had gained popularity in recent years and is being used as a first line treatment option for refractory GERD before surgical salvage [8]. Patients with sliding hernia more than 2 cm, severe reflux esophagitis (Los Angeles classification grade C/D), erosive esophagitis despite optimal PPI therapy, and primary extraesophageal conditions such as asthma should be excluded from this procedure [39]. 

## 5. Conclusions

Gastroesophageal reflux disease (GERD) is an increasing prevalent clinical condition affecting a significant portion of the population. This increase in incidence may well be associated with the awareness amongst medical practitioners and more efficient diagnostic techniques as well as other lifestyle factors. However, with the advent of minimally invasive procedures such as the various endoluminal techniques, there is now an increased array of management options available in addition to the traditional drug therapy and surgery. Current endoscopic intraluminal procedures gaining popularity include endoscopic fundoplication and radiofrequency ablation. These and any future minimally invasive endoscopic procedures certainly would be welcomed in addition to the management of GERD and would hopefully help to further alleviate the suffering of many GERD patients. 

## Figures and Tables

**Table 1:** Anti-reflux endoluminal therapies.

(1) Endoscopic fundoplication or suturing techniques	
EndoCinch	A landmark procedure approved by FDA in 2000 till today but the durability is poor even after enhanced modification
NDO PLICATOR	A full-thickness suturing transmural plicator to address the weakness of EndoCinch with successful creation of a more effective mechanical barrier but had been retrieved from market due to the company's poor financial performance
Esophyx	This transoral incisionless fundoplication (TIF) has the advantage that it can reduce hiatal hernia up to 2 cm, which is often not possible with other anti-reflux endoluminal therapies. Serious complications such as esophageal perforation and postoperative bleeding were reported

(2) Injection/implantation techniques	

Gatekeeper reflux repair system and Enteryx	(1) Creation of an anti-reflux barrier by a bulking effect at LES which included injection of bovine dermal collagen, Teflon, polymethylmethacrylate microspheres (Plexiglas), and polytetrafluoroethylene (Polytef) with no remarkable benefits (2) They were removed from the market due to unsatisfactory benefit from symptoms control or objective measurement of anti-reflux properties and various degrees of complications

(3) Radiofrequency ablation	

Stretta system	(1) It was first introduced in 2000 and utilizes an inflatable balloon-mounted device that introduces 4 electrodes at the LES with RFA energy delivered under controlled temperature to produce a coagulation inflammation, necrosis, and fibrosis. The technology has refined the recommended dosage to avoid serious complications (2) It had gained popularity in recent years and is being used as a first line treatment option for refractory GERD before surgical salvage

FDA: food and drug administration; TIF: transoral incisionless fundoplication; LES: lower esophageal sphincter; RFA: radiofrequency ablation; GERD: gastroesophageal reflux disease.
